# Aerobic degradation of crude oil by microorganisms in soils from four geographic regions of China

**DOI:** 10.1038/s41598-017-14032-5

**Published:** 2017-11-01

**Authors:** Qinglong Liu, Jingchun Tang, Kai Gao, Ranjit Gurav, John P. Giesy

**Affiliations:** 1 College of Environmental Science and Engineering, Nankai University, Tianjin, 300071 China; 2 Tianjin Engineering Center of Environmental Diagnosis and Contamination Remediation, Tianjin, 300071 China; 3 Key Laboratory of Pollution Processes and Environmental Criteria (Ministry of Education), Tianjin, 300071 China; 4 Tianjin Academy of Environmental Sciences, Tianjin, 300191 China; 5 Toxicology Centre, University of Saskatchewan, Saskatoon, Saskatchewan Canada; 6 Department of Veterinary Biomedical Sciences, University of Saskatchewan, Saskatoon, Saskatchewan Canada; 7 School of Biological Sciences, University of Hong Kong, Hong Kong, SAR China; 8 State Key Laboratory of Pollution Control and Resource Reuse, School of the Environment, Nanjing University, Nanjing, People’s Republic of China; 9 Department of Biology, Hong Kong Baptist University, Hong Kong, SAR China

## Abstract

A microcosm experiment was conducted for 112 d by spiking petroleum hydrocarbons into soils from four regions of China. Molecular analyses of soils from microcosms revealed changes in taxonomic diversity and oil catabolic genes of microbial communities. Degradation of total petroleum hydrocarbons (TPHs) in Sand from the Bohai Sea (SS) and Northeast China (NE) exhibited greater microbial mineralization than those of the Dagang Oilfield (DG) and Xiamen (XM). High-throughput sequencing and denaturing gradient gel electrophoresis (DGGE) profiles demonstrated an obvious reconstruction of the bacterial community in all soils. The dominant phylum of the XM with clay soil texture was *Firmicutes* instead of *Proteobacteria* in others (DG, SS, and NE) with silty or sandy soil texture. Abundances of alkane monooxygenase gene *AlkB* increased by 10- to 1000-fold, relative to initial values, and were positively correlated with rates of degradation of TPHs and n-alkanes C13-C30. Abundances of naphthalene dioxygenase gene *Nah* were positively correlated with degradation of naphthalene and total tricyclic PAHs. Redundancy analysis (RDA) showed that abiotic process derived from geographical heterogeneity was the primary effect on bioremediation of soils contaminated with oil. The optimization of abiotic and biotic factors should be the focus of future bioremediation of oil contaminated soil.

## Introduction

Bioremediation is the most efficient, economical way to deal with contamination of soils by petroleum hydrocarbons (PHs). It also does not generate toxic metabolites. Thus, it has been widely accepted and used for remediation of areas that have been contaminated long-term with petroleum^1^. Besides the presence of PHs, the structure of communities of microbes capable of degrading PHs also depends on properties of soils in different geographic regions (i.e., physical-chemical as well as biological characteristics). Examples of this variability are *Actinobacter* sp., and *Bacteroidete* sp., which were the dominant alkane-degrading bacteria in soils of the Huabei Oilfield^2^, while the community on King George Island was composed mainly of *Rhodococcus* sp., *Acinetobacter* sp., *Mycobacterium* sp., *Gordonia* sp., and *Aeromicrobium* sp.^3^. *Pedobacter* sp. and *Mycobacterium* sp. were the major bacteria capable of degrading alkanes in areas where PHs are produced in the Karamay Oilfield^4^, while dominant alkane-degrading bacteria in soils from the Shengli Oilfield were *Alcanivorax* sp. and *Acinetobacter* sp.^5^. Based on profiles of phospholipid fatty acid, populations of gram-positive bacteria were significantly larger in petroleum contaminated upland soils than paddy fields^6^.

Previously, in contaminated ecosystems, relationships between dominant populations of microbes and intensity of contamination with PHs have been studied. Microorganisms inhabiting locations contaminated with PHs often contain enzymes that can degrade oil and thus might be of interest for scientific and industrial applications^7,8^. Recently developed molecular biotechnology, involving direct isolation of nucleic acids from environmental samples followed by denaturing gradient gel electrophoresis (DGGE), real-time quantitative polymerase chain reaction (qPCR) and high-throughput DNA sequencing (HTS) have been used to determine diversity of endogenous microorganisms^9^. Due to their highly conserved genetic topologies, genes capable of degrading oil have been used as biomarkers for determination of abundances and diversities of oil-degrading microorganisms from various environments^10,11^. Recently, real-time PCR initially developed for quantifying expressions of genes, has also been applied in microbial ecology and has become the most popular alternative for measurement of 16S rRNA for genes to describe specific microbial populations^12^. Alternatively, HTS using Illumina HiSeq technology provides a robust method to detect genome sequence information of all autochthonal organisms and describe their communities more comprehensively^13^.

Microbes capable of degrading PHs can operate on a wide range of metabolic substrates, and can even be adapted to polar regions^14^. Diverse microbial flora can degrade PHs anaerobically or aerobically by use of a variety of enzymes encoded by key functional genes, such as alkane hydroxylase genes, ring-hydroxylating dioxygenase α-subunit (RHDα) genes^15^, alkylsuccinate synthase gene (*assA*) and benzylsuccinate synthase gene (*bssA*)^16^. The main components of PHs are saturated aliphatic and aromatic hydrocarbons. During the initial activation step of aerobic aliphatic hydrocarbon metabolism, a non-heme integral membrane alkane monooxygenase encoded by *AlkB* gene is expressed. The *AlkB* can transfer electrons from nicotinamide adenine dinucleotide phosphate (NADPH) to PH substrates in the presence of the cofactors, rubredoxin (*AlkG*) and rubredoxin reductase (*AlkT*)^17^. Alternatively, during aerobic degradation of PAHs of smaller molecular masses, naphthalene dioxygenase systems encoded by *Nah* gene and phenol monooxygenase encoded by the *Phe* gene during the initial phase add single or two atoms of molecular oxygen to aromatic rings, respectively. Thus, quantification of these hydroxylase genes is interesting and important for assessment of potential for bioremediation by microbial communities of soils contaminated by PHs^18^.

In this study, laboratory-scale microcosms were used to study process and microbial responses to PHs spiked into soils from different geographic regions of China. Dynamic changes in genera within microbial communities and functional genes responsible for degradation of PHs were monitored using advanced techniques including DGGE, real-time quantitative PCR and high-throughput DNA sequencing. The studies were focused on enumeration of *AlkB*, *Nah* and *Phe*, which are thought to be responsible for hydroxylation of saturated aliphatic and aromatic hydrocarbons.

## Results

### Soil physical-chemical properties

The major physical-chemical characteristics of soils collected from different geographic sites are shown (Table 1). There was a great divergence in pH and salinity between soils from the north and south of China. In the north of China, DG and SS soil had pH values greater than 8 and salinities greater than 17 g kg^−1^. However, pH was 5.27 in XM soil which was from in the south of China. NE exhibited a pH of less than 7. DG and NE contained greater amounts of TOC exceeding 17 mg g^−1^, and a similar particle size distribution of about 5.6% clay, 77% silt and 17.5% sand as average value. However, SS and XM contained lesser amounts of TOC of less than 6 mg g^−1^. Sand content of soils from SS was greatest with a value of 96%, while contents of clay and silt were least at 0.56% and 3.37%, respectively. XM soil had the greatest clay and silt contents, in contrast to the least sand.

**Table 1:** Physical-chemical properties of soils.

Location	pH	Salinity (g/kg)	TOC (mg/g)	Particle size (%)		
				Clay (<2 μm)	Silt (2–50 μm)	Sand (>50 μm)
DG	9.23	17.657	20.82	5.8	78.4	15.8
SS	8.73	36.783	2.25	0.56	3.37	96.07
NE	4.78	3.725	17.62	5.47	75.41	19.12
XM	5.27	0.75	5.28	10.27	81.08	8.65

DG: Dagang Oilfield; SS: Sea Sand; NE: Northeast China; XM: Xiamen.

### Degradation of petroleum hydrocarbons in different soil microcosms

**Figure 1:**
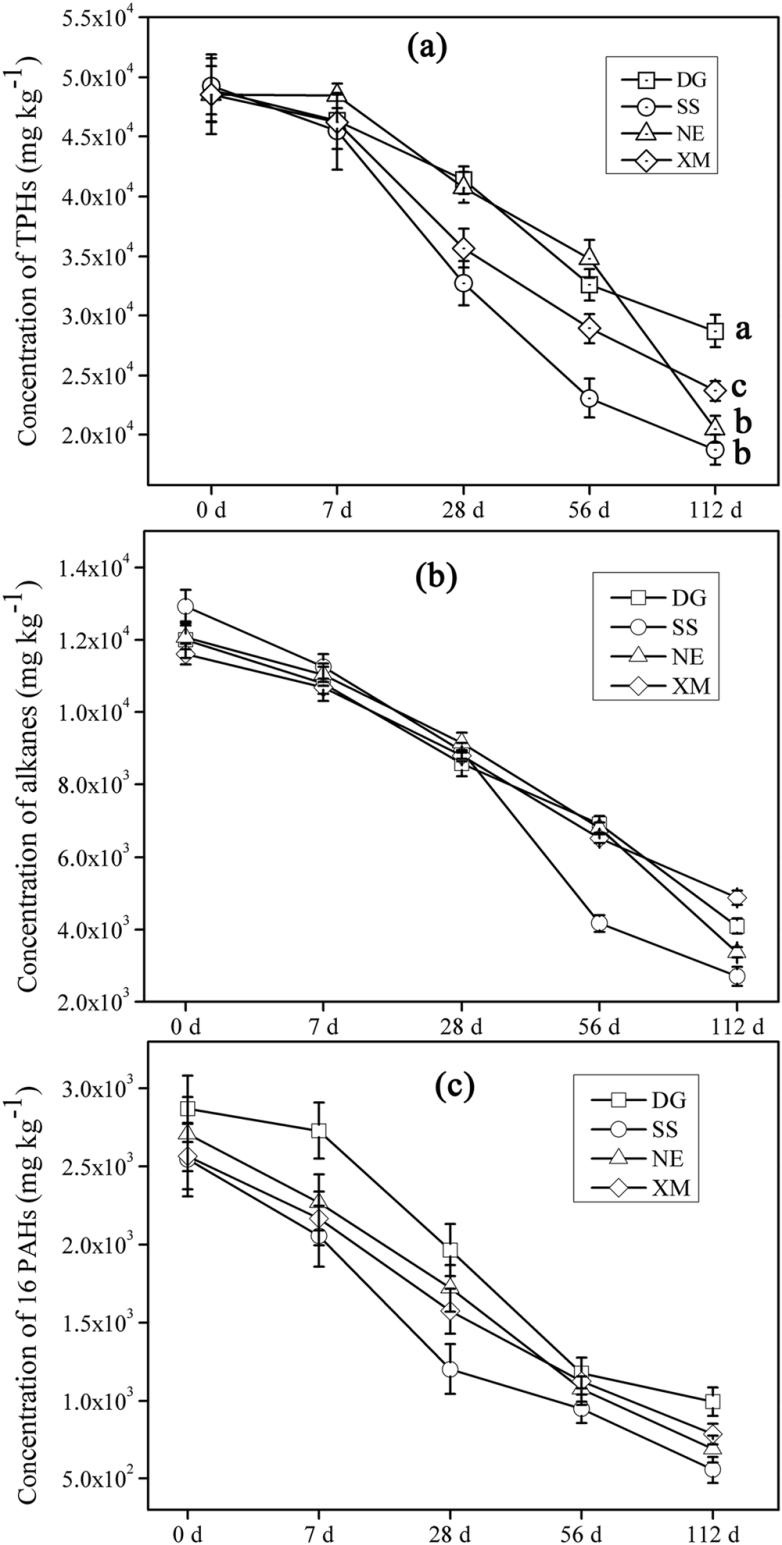
Concentrations of petroleum hydrocarbons (PHs) in soils from four geographic regions. (**a**) Concentrations of total petroleum hydrocarbons (TPHs) in microcosm soils; (**b**) Concentration of n-alkanes (C8-C40) in microcosm soils; (**c**) Concentration of 16 ΣPAHs in microcosm soils. Statistically significant differences between the sampling sites (p < 0.05) are indicated by the different letters in each column. Error bar represent standard deviation (n = 3).

Gravimetric estimation of total concentrations of PHs in soils initial and after various durations of incubation of microcosms was done to determine the degradation of PHs (Fig. 1a). All microcosms containing various soils resulted in approximately 50% loss of TPHs via degradation compared to sterile soils. Microbial flora in SS and NE soils exhibited greater potential to degrade TPHs, with maximum reduction of TPHs to 1.87 × 10^4^ (SS) and 2.04 × 10^4^ mg kg^−1^ (NE) after 112 d, from initial concentrations of 4.92 × 10^4^ (SS) and 4.85 × 10^4^ mg kg^−1^ (NE) (t = 0 d), which represented 62% and 58% of TPHs degradation, respectively. Residual concentrations of n-alkanes (C8-C40) and PAHs were also determined by use of GC-MS (Fig. 1b,c and Supplementary Figure S1). SS soils exhibited the greatest ability to degrade C8-C40 compounds and concentrations of C8-C40 alkanes were reduced from 1.29 × 10^4^ (t = 0 d) to 0.27 × 10^4^ mg kg^−1^ (t = 112 d), with degradation as great as 79%. Alkanes (C13-C30) which were the predominant alkanes were mineralized from 1.07 × 10^4^ to 1.76 × 10^3^ mg kg^−1^ by microbes in SS showing the highest mineralization of 84%. XM soil exhibited the least degradation of alkanes C8-C40 and C13-C30 with 58% and 59%, respectively. Similarly, degradation of PAHs in the four soils showed similar trends with SS showing the highest PAHs degradation up to 78%. Degradation of tricyclic and tetracyclic Ahs, which were the main components of 16 ΣPAHs, were as much as 78% and 79% in SS, respectively. SS showed the greatest biodegradability of PAHs compared to other soils especially soil DG, of which the PAHs degradation was only 65%. The degradation of tricyclic and tetracyclic AHs in DG were also least with 62% and 68%, respectively.

### Correlations between biodegradation of PHs and soil properties

**Figure 2:**
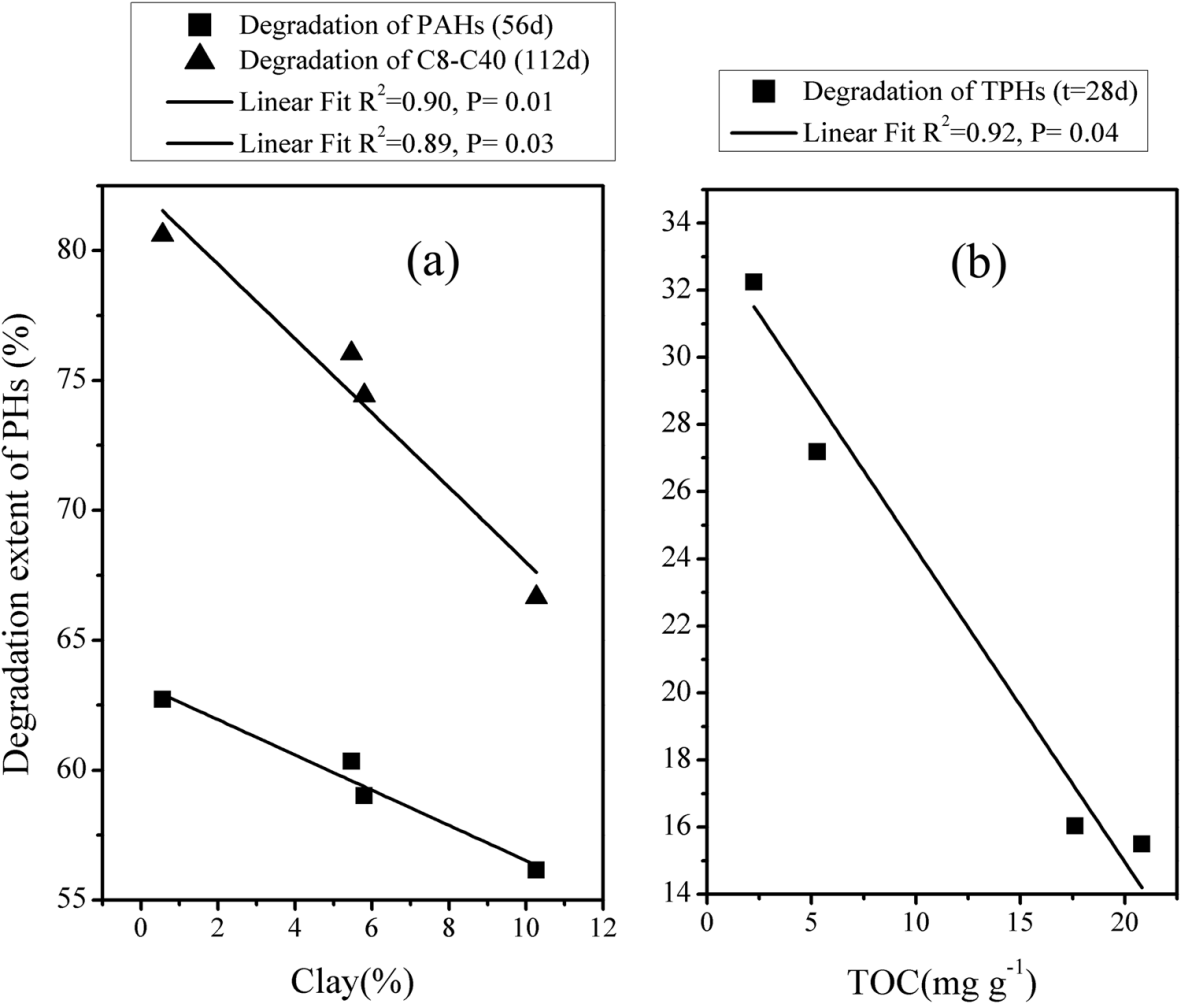
Pairwise correlations between soil properties and degradation of PHs. (**a**) Pairwise correlations between clay content and PHs degradation; (**b**) Pairwise correlations between TOC contents and degradation of PHs.

Biodegradation of PHs was significantly and negatively correlated with clay contents of soils. Degradation of total alkanes over 112 d was negatively correlated with clay content of the soils (R^2^ = 0.90, *p* = 0.01). Rates of degradation of PAHs over 56 d was also significantly and negatively correlated (R^2^ = 0.89, *p* = 0.03) (Fig. 2a) with clay content of soils. During the initial phase (t = 28 d) of the study, there was also a significant negative correlation (R^2^ = 0.92, *p* = 0.04) between TOC content and degradation of TPHs (Fig. 2b).

### High throughput sequencing

Based on partial 16 S rRNA sequences, numbers of bacteria in soils from four geographic regions ranged from 5777 sequences to 13854 sequences and contained 94–247 operational taxonomic units (OTUs) based on 97% nucleotide sequence identity (Supplementary Table S1). Results of a cluster analysis and relative abundance of taxonomic assignments for all samples at initial (t = 0 d) and last (t = 112 d) are shown (Supplementary Figure S2). Initially, *Proteobacteria* was the dominant phylum in DG and SS soils with relative abundances of 34.31% and 86.56%, respectively. However, the dominant microbial phylum in NE and XM were both *Firmicutes* of which the relative abundances were 36.90% and 78.67%, respectively. Over the duration of cultivation in the microcosms, the abundance of *Proteobacteria* increased in all the soils. Increases were from 34.31% to 73.56% in DG; from 86.56% to 97.65% in SS; from 21.48% to 74.62% in NE; from 12.65% to 59.53% in XM). In contrast, abundances of *Actinobacteria*, *Bacteroidetes*, and *Firmicutes* decreased in all soils.

Detailed changes in proportions of microbial genera detected by HTS are given in a heat map (Figure S3). In DG soil, the relative abundance of *Marinobacter* increased from 11.60% (t = 0 d) to 22.65% (t = 112d). In SS soil, the genus *Pseudomonas* was the primary alkane-degrading bacterium of which relative abundance decreased from 68.73% to 39.30%, while the proportion of *Acinetobacter* increased from 10.80% to 21.64% after the 112 d of cultivation. In NE soil, the abundance of *Rhodanobacter* increased from 1.26% to 36.24% to be the dominant genus. In XM soil, the relative abundances of dominant genera such as *Lactococcus* (belonging to *Firmicutes*) decreased from 39.78% to 16.96%. *Sphingobium* (*α-Proteobacteria*) and *Burkholderia* (*β-Proteobacteria*) were significant populations of microbes with the ability to degrade PHs of which the relative abundances were 14.03% and 13.51%, respectively.

### Characterization of microbial community by DGGE fingerprints

**Figure 3:**
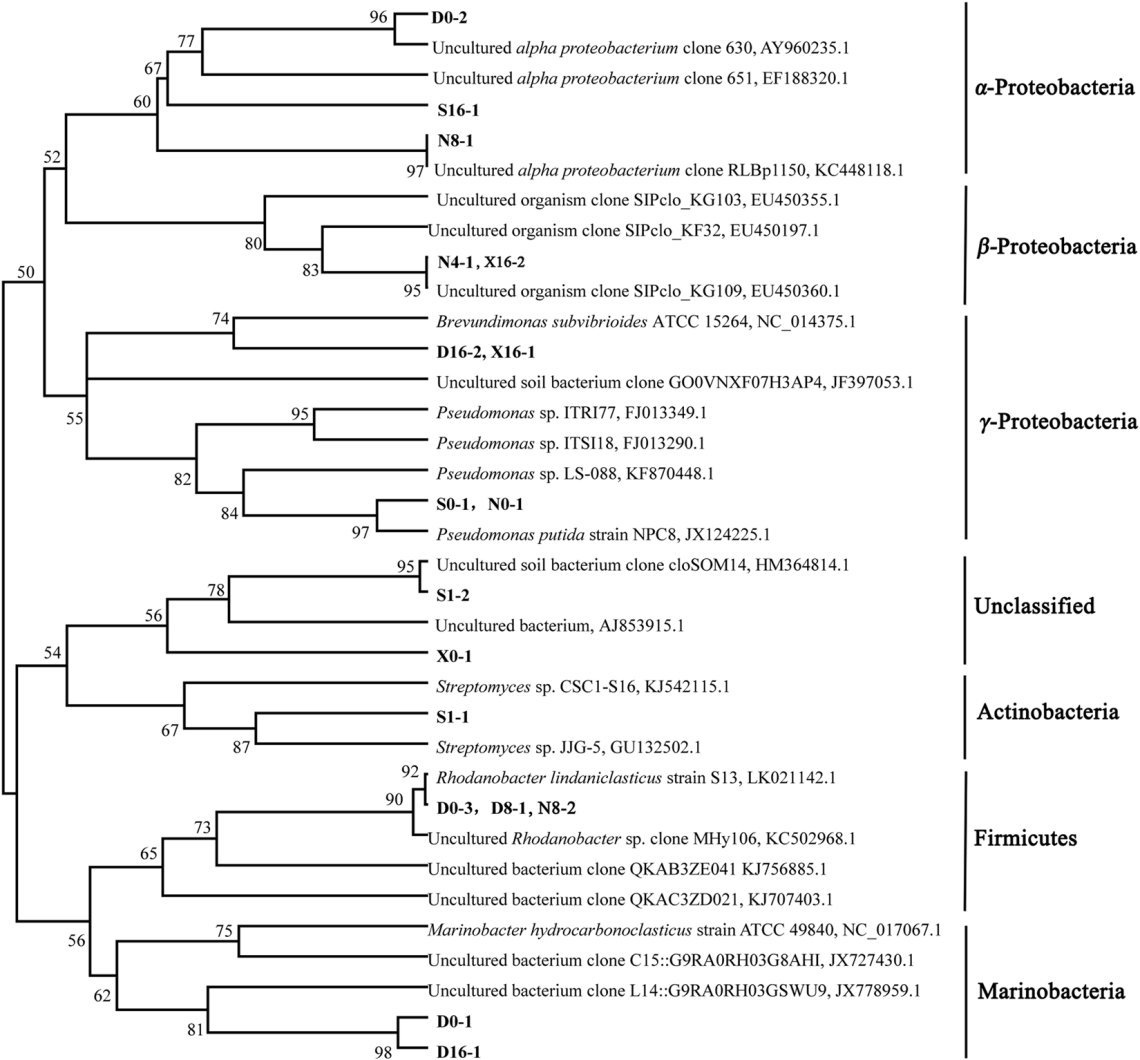
Phylogenetic tree constructed based on 16S rRNA sequences using neighbor-joining methods. Sequences were referred from NCBI database, and the corresponding GenBank accession numbers were labeled after the name of the strains. Associated taxa were clustered in the bootstrap test (1000 replicates), and the bootstrap values were greater than 50%.

PCR-DGGE analysis was performed to determine shifts in patterns of relative abundances of bacterial populations in soils from different soil environments which had been amended with crude oil (Supplementary Figure S4). The sequences of V3 regions of 16S rRNA gene from gel were compared to the GenBank and homologous strains are shown (Supplementary Table S2). Partial sequences of 16S rRNA from genes 338 f to 518r were compared to the GenBank database by use of the Basic Local Alignment Search Tool (BLAST). Alignments were performed by Clustal X (version 1.81) with default values. A phylogenetic tree was constructed using the neighbor-joining method in the Mega 5.0 program (Fig. 3). A sequence having similarity less than 95% found in the database suggested a novel new genus of bacteria.

Sequencing bands in DGGE profiles and the phylogenetic tree demonstrate the shift in relative numbers of OTUs in microbial communities. In DG soils, the uncultured *Rhodanobacter* sp. sequenced from D0-3 band was the native bacterium, which gradually disappeared after treatment for 28 d, however, appeared again after 56 d (D8-1). *Brevundimonas* sp. (band D16-2) was the dominant species among microbial communities under this study. DNA sequences from bands D0-1 and D16-1 were identified as *Marinobacter* sp. During initial and final phases of incubation, *Marinobacter* sp. showed a great abundance of community population. *Pseudomonas* sp. sequenced from the S0-1 band from SS soil exhibited an intense band pattern on the DGGE gel throughout incubation. A strain of uncultured bacterium (X0-1) was initially the dominant autochthonal microorganism. However, culturable *Brevundimonas* sp. (X16-1) exhibited a greater abundance after 28 d. The Shannon-Weiner index was used to investigate changes in diversity of microbial communities among the four soils (Supplementary Table S3). The Shannon-Weiner index decreased after 7 days of incubation in all soils and then increased again after 28 days and decreased again at the late period of the process. While the Shannon-Weiner index values were different with the following order at initial: NE > DG > XM > SS, which was changed to the following order in the end: DG > SS > XM > NE.

### Abundances of typical oil catabolic genes

**Figure 4:**
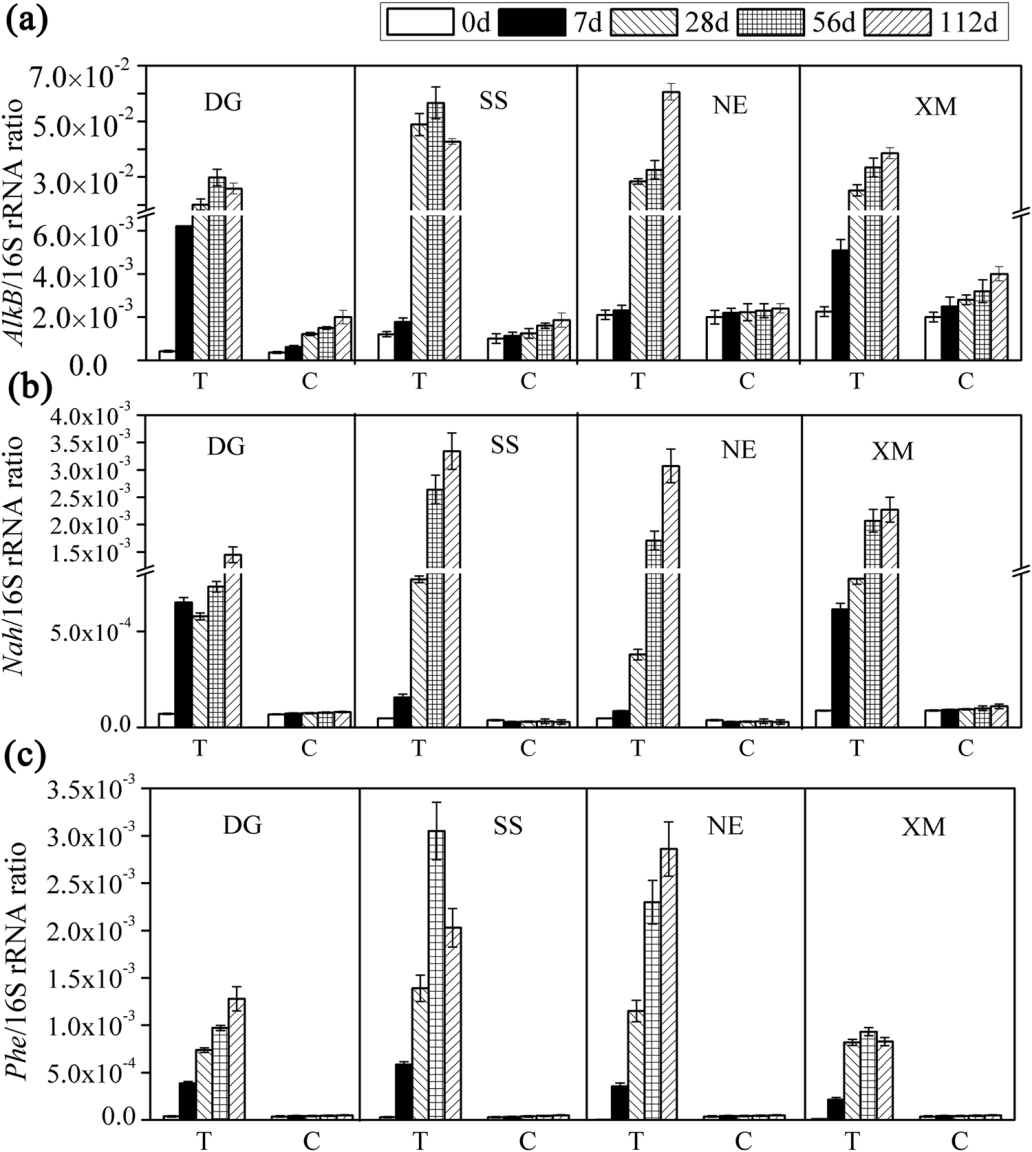
Relative abundances of oil-degrading genes in four soils spiked with crude oil. Abundances of genes were normalized to the total 16S rRNA genes. Note: C represents CK without addition of petroleum hydrocarbons; T represents test groups with addition of petroleum hydrocarbons.

Relative abundances of metabolic genes normalized to total number of copies of 16S rRNA genes are shown (Fig. 4). Absolute copies of oil-degrading genes are listed in Supplementary Table S4. Relative abundances of genes capable of catabolism of PHs in soils changed significantly during incubation of soils in microcosms. Initially (t = 0 d), relative abundances of the *AlkB* gene in soils from the four geographic regions ranged from (4.22 ± 0.33) × 10^−4^ to (1.21 ± 0.12) × 10^−3^, while the *AlkB* gene accumulated during incubation. The maximum relative abundance of the *AlkB* gene in soil NE (6.06 ± 0.38) × 10^−2^ was observed on 112 d, followed by soil SS (5.67 ± 0.26) × 10^−2^ after 56 d. These values were 10 to 100-fold greater than those observed during initial stages of incubations of soils and consistent with greater degradation of alkanes in soils SS and NE.

Similarly, during incubation, the relative abundance of the *Nah* gene changed substantially in all four soils. An almost identical trend was observed for relative abundance of the *Phe* gene in soils NE and SS, which also increased 1000-fold from 10^−6^ to 10^−3^, whereas, the *Phe* gene increased from 10^−5^ to 10^−3^ in DG and XM. Results of statistical analysis (ANOVA) showed that relative abundances of degradation genes *AlkB*, *Nah*, and *Phe* were significantly different (*p* < 0.01) among the four soils and among sampling times, except for the *AlkB* gene in soils NE and XM after 56 d, and initial abundances of the *Nah* gene in soils SS and NE.

### Correlations between mineralization of petroleum hydrocarbons and indices of microbial communities among soils

**Figure 5:**
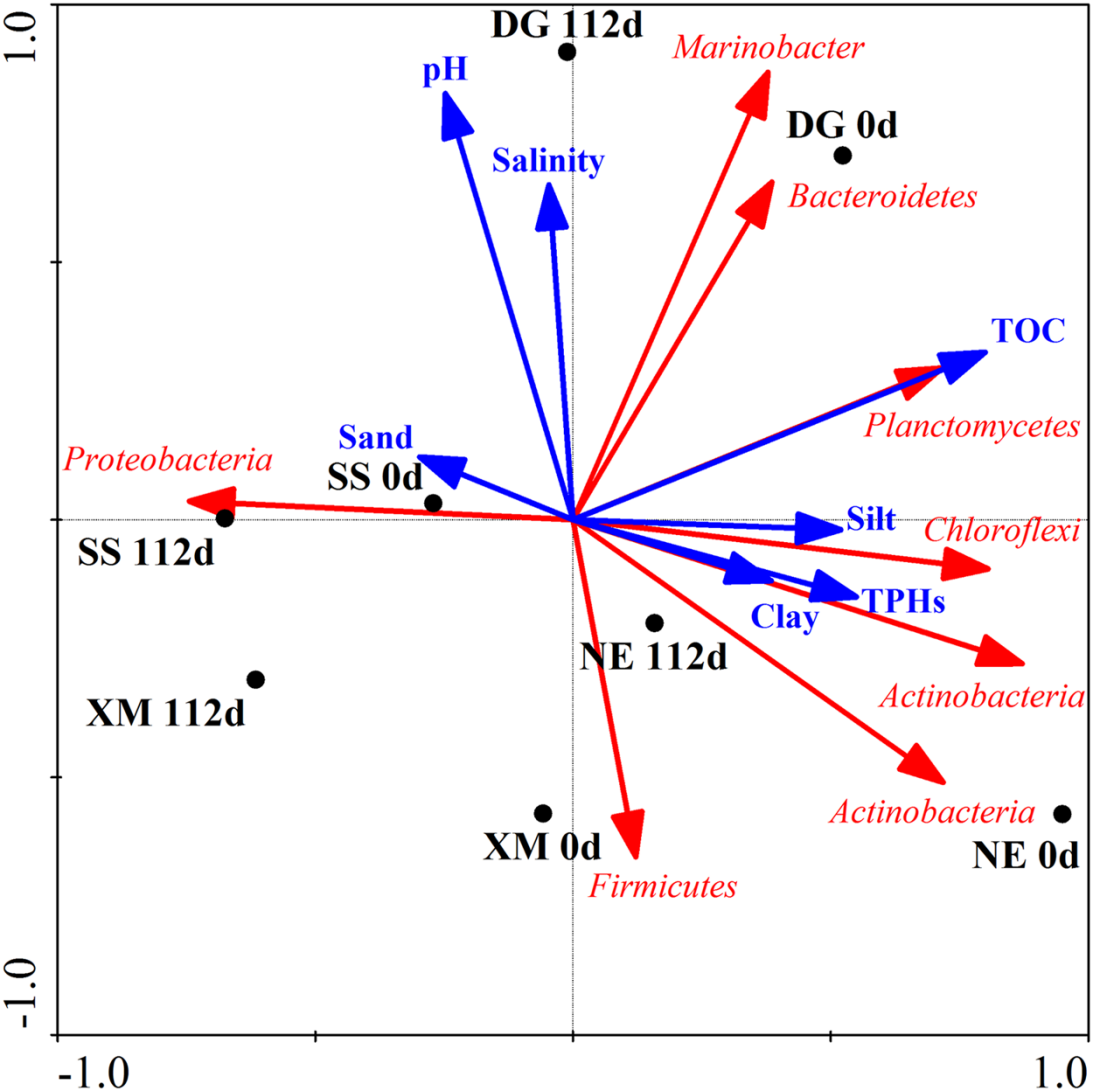
Redundancy analysis (RDA) biplot depicting the relationship between the bacterial communities and main physicochemical parameters of the four different geographic soil samples. Note: Solid circles represent the four different soil samples collected at initial and last experimental time. Environmental variables that significantly explained variability in composition of microbial communities were fitted to the ordination. Arrows indicate the direction and magnitude of environmental variables associated with the different bacterial phylum.

The results of which are presented here, significant positive correlations were observed between relative abundance of the *AlkB* gene and degradation of TPHs (R^2^ = 0.94, *p* < 0.05) as well as medium-short chain alkanes (C13-C30) (R^2^ = 0.85, *p* < 0.05) in all soils after 112 d (Supplementary Figure S5a). Positive correlation was also found between relative abundances of the *Nah* gene and mineralization of naphthalene (R^2^ = 0.88, *p* < 0.05) and total tricyclic PAHs (R^2^ = 0.81, *p* < 0.05) (Supplementary Figure S5b). Redundancy analysis (RDA) in Fig. 5 showed that soils DG, SS, NE and XM, which were collected from four geographic regions of China, varied greatly in physical properties and presence of microbial communities capable of degrading PHs.

## Discussion

Compared to the shorter microcosm cultivation used in previous studies, this study reached a stable stage of availabilities of alkanes and PAHs to microbes^19,20^. Physical properties and presence of microbial communities capable of degrading PHs varied among soils DG, SS, NE and XM, which were collected from four geographic regions of China, varied. Microbial flora in SS and NE soils exhibited greater potential to degrade TPHs as well as the PH components (16 ΣPAHs, alkanes C8-C40) than that in DG and XM soils. Bioavailability of petroleum hydrocarbons among soils, in part due to enrichments of various microbial communities which have been adapted to the specific environmental factors^21,22^.

Obvious changes in bacterial communities were observed, which demonstrated that there was no tendency for the bacterial community of the four different soil types to converge as a result of contamination with crude oil. The dominant genera of bacteria in DG, SS, NE, and XM were *Marinobacter*, *Pseudomonas*, *Rhodanobacter*, and *Lactococcus*, respectively after 112 d of cultivation. Dominant phylum of the XM with clay soil texture was *Firmicutes* instead of *Proteobacteria* in other sites (DG, SS, and NE) with silty or sandy soil texture. The dominant phylum in clay soils has been shown to be *Actinobacteria*, while that in sandy or sandy loam soil texture was *Proteobacteria* ^23^. A similar conclusion, based on divergence of microbial communities among six types of soil, for which textures ranged from clay to loam, and pH values ranged from 5.4 to 8.8 was reported^24^. DG soil, which had the greatest salinity, contained a great abundance of saline and alkaline-tolerant bacteria. *Marinobacter* has previously shown to be efficient at metabolism of alkanes^25^. Uncultured *Rhodanobacter* sp. was the native bacterium which gradually disappeared after treatment for 28 d, however, appeared again after 56 d. Previously, it has been shown that *Rhodanobacter* sp. was easily inhibited by PAHs, whereas it could degrade intermediate metabolites which enabled further degradation of hydrocarbons^26^. *Brevundimonas* sp., which was previously isolated and identified from bottom sludge in a crude oil tank and hydrocarbon-contaminated soil of Azzawiya oil refinery plant^27^ had a great accumulation in DG. *Pseudomonas* sp. was present in SS soil, which had the greatest capacity to degrade both PHs and alkanes. Previously, it had been reported that *Pseudomonas putida* GPo1 degraded alkanes by a monooxygenase enzyme encoded by the *AlkB* gene^28^. In NE soil, *Rhodanobacter* (*γ-Proteobacteria*) was the dominant genus which has been shown to have a large capacity to degrade various PAHs and play an important role in bioremediation of petroleum hydrocarbons^26^. *Sphingobium* (*α-Proteobacteria*) and *Burkholderia* (*β-Proteobacteria*) were significant populations of microbes with the ability to degrade PHs in XM soil. Previous studies have found that both *Sphingobium* and *Burkholderia* can mineralize the PAHs, especially for BTEX^29,30^. A new strain of uncultured bacterium was initially the dominant autochthonal microorganism in XM soil. Results of previous studies using clone libraries, hybridization probes and other molecular biological techniques have shown that new genotypes PHs-degrading genes are widely contained in native microorganisms in sea water, and marine sediments and soils of oilfield that are contaminated with PHs^31,32^.

Abundances of catabolic genes represent the degradation potential of microflora, and can be used as an indicator of the community’s response to bioavailable hydrocarbons. Therefore, differences in abundances of catabolic genes are assumed to result from divergent accumulation of bacterial populations within biogeographic sampling sites. These values of relative abundance of *AlkB* gene were 10 to 100-fold greater than those observed during initial stages of incubations of soils and consistent with greater rates of degradation of alkanes in soils SS and NE. Since each bacterial cell contained 3-4 copies of *AlkB* gene based on the previously described analytical approach^33^, the total population of alkane-degrading bacteria was calculated to be between 4.27% and 5.67% in SS, and between 2.85% and 6.06% in NE during the period from 56 d to 112 d. These values are similar to the proportion of bacteria reported to be capable of degrading alkanes reported by Xu *et al*.^5^ who determined the alkane-degrading bacteria to be between 4.7% and 7.4% in soils of the Shaozhuang oil and gas field soil, and Shengli Area, China, respectively. The proportion of alkane-degrading bacteria in soils from DG and XM were 2.98% and 3.86% in their respective maxima which were less than those in SS and NE. Absolute abundances of *AlkB* in four soils were similar to those in soils from the Daqing and Karamay Oil Fields, China, which had ranges of 10^6^–10^7^ and 10^5^–10^7^ copies/g, dm soil, respectively^4^, but greater than that in sediments from the Timor Sea Australia that had been contaminated with oil, where the values ranged from 1.1 × 10^5^ to 2.9 × 10^5^ copies/g, dm soil^34^. Aromatic hydrocarbon-degrading bacteria expressing the *Nah* gene in all soils showed same range of relative abundance as that of bacteria that are capable of hydroxylating PAHs quantified previously^18^. An almost identical trend was observed for relative abundance of the *Phe* gene in soils NE and SS. *Phe* encodes for the enzyme phenol monoxygenase, which can further mineralize intermediates in degradation of monoaromatic hydrocarbons^35^. Abundances of the *Nah* and *Phe* genes were used as indicators for estimating the PAH-degradation potential of aromatic oxygenase present in autochthonic bacterial communities^36,37^.

Bioremediation of soils contaminated with PHs depends on abundances of metabolic genes, which can encode for hydroxylase enzymes. In the study, except the higher degradation extent of PHs found in SS, due to the same order of relative abundance of three degradation genes, which had a range of 10-1000-fold increase, similar degradation was observed in other soils. While the microbial responses to oil contamination were different at the same site under the different oil exposure time, RDA analysis demonstrated that the soil properties were the dominant effect factors on the microbial communities and TPHs degradation. In detail, the pH and salinity were restrictive factors on the accumulation of degradation microbes in DG soil, and only the saline and alkaline-tolerant bacteria such like *Marinobacter* account for the great abundance. pH has been proved the important factor in determining the composition of microbial community especially in saline lake and tailing dam soils^38,39^. Particle size was the dominant determining factor of the microbial degradation potential in NE, XM, and SS soil. NE and XM, which contained greater fractions of clay and silt, exhibited lesser PHs degradation. Results of a previous study demonstrated that PHs absorbed on clay minerals by hydrogen bonding and electric dipole van der Waals forces were recalcitrant to degradation by soil microorganisms^40^. Soil SS, which contained a greater proportion (96.07%) of sand exhibited the maximum potential to degrade PHs. Soils containing greater contents of sand can provide the adequate oxygen to aerobic microbes which so that exhibited greater PHs degradation^41^. During the initial stage, TPHs were more likely to be degraded by indigenous microbes in soils with lesser concentrations of TOC. Meanwhile, TOC content was the key factor affecting the magnitude and relative compositions of microbial communities, especially *Planctomycetes* and *Proteobacteria*. Results of previous studies have demonstrated that some carbon sources derived from non-petroleum hydrocarbons (biogenic and anthropogenic carbonaceous organic compounds) are more readily metabolized by microorganisms which results in competitive metabolism with PHs^42^. Nevertheless, due to the accumulation of specialized PHs degrading microorganisms, as well as formation of co-metabolic pathways on PHs and non-petroleum hydrocarbon degradation by microbes^43^, the negative correlation between TOC content and degradation extent of PHs was not observed after initial stages of incubations of soils.

Relative abundances of metabolic genes are good biomarkers to evaluate potential of PHs to be degraded in soils. In addition, heterogeneity of physical-chemical properties of soils contaminated with PHs complicates the prediction of PHs mineralization. In silty soils (silt content: 75.41~78.4% wt), halophilic *Marinobacter* were the dominant bacteria capable of degrading PHs at greater pH (>9.0) and salinity (>17.65 g kg^−1^). However, indigenous bacteria capable of degrading PHs, such as *Rhodanobacter* prefer to accumulate in soils of lesser pH (<4.8) and salinity (>3.8 g kg^−1^). *Pseudomonas* played an important role in PHs metabolism in sea sand with higher pH (>8.7) and salinity (>36.78 g kg^−1^) and 96.07% wt of sand content, while *Firmicutes* had the greatest abundance at lower pH (<5.3) and salinity (<0.8 g kg^−1^) soil with 10.27% wt of clay content. Therefore, research on structures of microbial communities and abundances of genes is critical to predict favorable conditions for bioremediation of soils contaminated with crude oil.

## Materials and Methods

### Sampling and microcosm simulation

**Figure 6:**
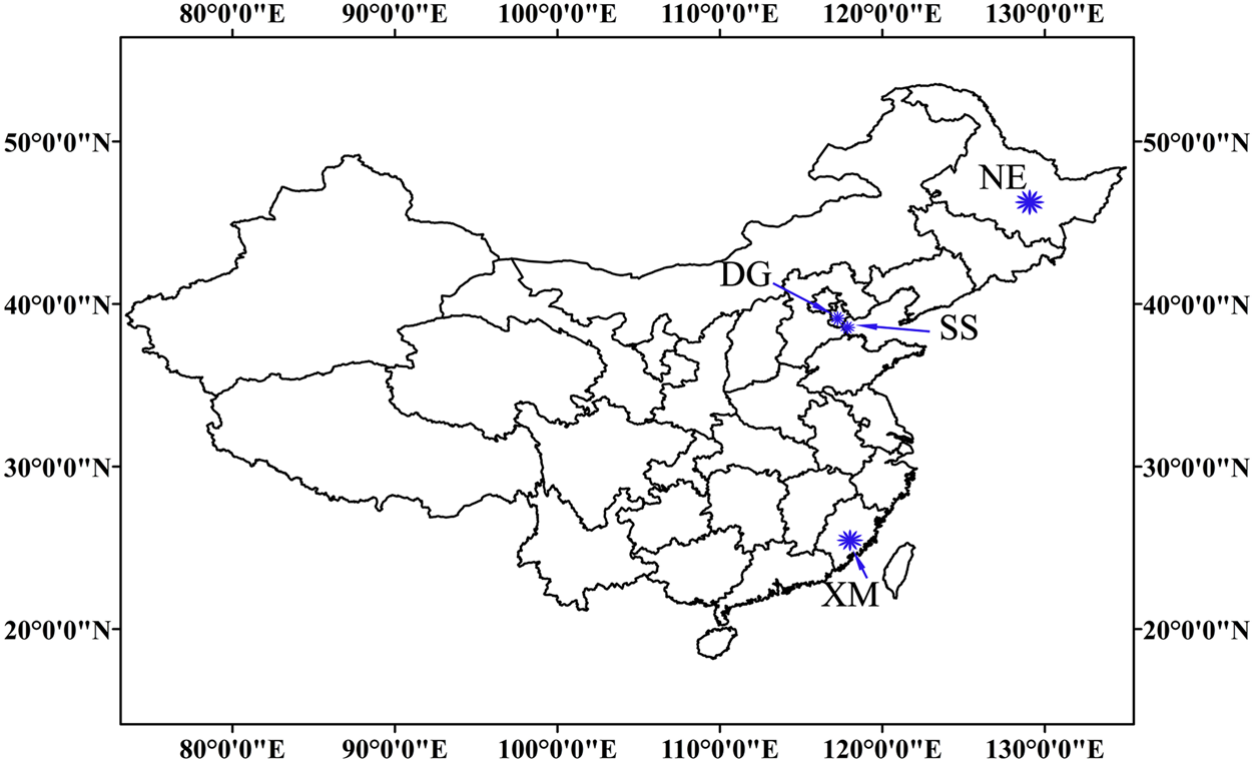
Locations from which soils were collected from four regions of China. DG: Dagang Oilfield; SS: Sand of Bohai Sea; NE: Northeast of China; XM: XiaMen. The figure map was generated by using software ArcGIS 10 (Environmental Systems Research Institute, Inc. Redlands, US). http://www.esri.com.

Soils which had not been contaminated with PHs (less than limits of detection) were collected from four geographic locations in China, Dagang Oilfield, Sand of Bohai Sea, Xiamen, and Northeast China (Fig. 6). The Dagang Oilfield is located in a coastal saline area, near Tianjin, China where soils contain greater salinity and alkalinity. The Bohai Bay Rim is one of the most important offshore bases for exploration and transportation of oil. Northeast China is famous for its fertile black soil, and is one of the three black earth terrains in the world. Red earth in Xiamen is typically acid and orthic ferralsols with unsaturated basicity. Soils were collected from a depth of 0–20 cm using sterile shovel. Samples of soil were air-dried prior to quantification of PHs and other physical and chemical properties. Samples of soil for analysis of diversity and abundance of microbial catabolic genes were thoroughly homogenized to obtain a uniform sample and stored at −20 °C.

To study effects of types of soils and microbial communities on degradation of crude oil, soil was incubated in 850 mL volume polytetrafluoroethylene equipment (Inner diameter 8.5 cm; Height 15 cm) containing 500 g soil to which 5% (mass) crude oil (provided by No. 1 Oil Production Plant, PetroChina Dagang Oilfield Company, see Table 2) by use of previously described methods^44^. Original composition of the crude oil is shown (Table S5 of Supporting Information). Soils for the study of abiotic factors influence were sterilized by autoclaved sterilization. The blank, “control” was maintained without addition of any petroleum contaminants. The total volume of sterile, deionized water was determined and added to DG, SS, NE, and XM soils as per their water characteristic to achieve an equivalent matric potential of 40, 22, 34 and 35 kPa, respectively (32.2%, 39.6%, 30.5%, and 31.8% water content, respectively). Microcosms were established in triplicate and incubated for 112 days in dark condition at the room temperature. Soils were allowed to equilibrate after oil spiking, and then experiments were initiated. Approximately 10 g of soil were collected by use of sterilized shovel at the sampling time of 0, 7, 28, 56, and 112 d during microcosm process. Each treatment (soil type) was carried out in triplicate.

**Table 2:** Original composition and element contents of the crude oil (provided by PetroChina Dagang Oilfield Company).

Components of crude oil (%)	Saturate hydrocarbons	Aromatic hydrocarbons	Resin	Asphaltene	
	57.2	28.6	10.7	3.5	
Element contents (%)	C	H	S	N	O
	85.67	13.40	0.12	0.23	—

### Determination of physical-chemical properties of soil

The pH was determined by pH meter (Sartorius, Germany) in a suspension of 1:5 (soil:deionized water). Salinity was determined gravimetrically^45^. In brief, a suspension of soil was heated in a water bath at 100 °C to dryness, and 10% H_2_O_2_ was added as oxidant subsequently for three times. Residual salt residual was then weighted and salinity of the soil calculated. Analyses of particle sizes of soils were determined via BT-9300S laser particle size analyzer (Dandong Bettersize Instruments Ltd. Liaoning province, China). Total organic carbon was measured through Multi N/C 3000 (Analytik Jena AG, Germany).

### Determination of petroleum hydrocarbons

Concentrations of total petroleum hydrocarbons (TPHs) in soils were determined gravimetrically. A five-gram aliquant of moist soil (after deduction of moisture content) was Soxhlet-extracted for 12 h with 120 mL dichloromethane at 54 °C^46^. Extracts were concentrated to dryness using rotary evaporator, and masses of TPHs were measured by gravimetric method^47^. All extractions and quantifications were performed in triplicate.

PAHs and saturated hydrocarbons (SHs) were separated by silica gel-alumina column and quantified by use of a model Agilent 7890 gas chromatograph connected to a 5975 Agilent HP mass spectrometer (Agilent, CA, USA) as per previous study^48^. Mixtures of 16 target PAHs on the EPA priority pollutant list (see Supplementary Figure S6) and 33 target n-alkanes (C8-C40) were used as standards for external determination of components of extracts^49^. Extraction efficiencies of PAHs and SHs were ranged from 82% to 105%.

### Molecular analysis

#### High throughput sequencing of partial 16S rRNA genes

Genomic DNA extraction was performed by a ZR microbe DNA MiniPrep™ kit (Zymo Research, CA, USA) according to the manufacturer’s protocol. Extracted DNA was checked by 1.5% agarose gel electrophoresis. To determinate changes in microbial communities during bioremediation of soils contaminated with PHs, from 0 d to 112 d, the V4-V5 fragment of the 16S rRNA gene was amplified with a set of primers 515f (GTG CCA GCM GCC GCG GTAA) and 907r (CCGTCAATTCMTTTRAGTTT) following a previously described protocol^50^. Amplicons were sequenced by using the Illumina HiSeq. 2000 platform (Majorbio co., Shanghai, China). Sequences were excluded from the analysis if the read length was less than 150 bp. Corrected sequences were then processed using the UPARSE pipeline^51^ within QIIME (Quantitative Insights Into Microbial Ecology), and operational taxonomic units (OTUs) were defined at 97% nucleotide similarity. Sequences for each OTU and the Ribosomal Database Project (RDP) classifier were used to assign taxonomic data^50^. Sequences of amplicons from Illumina HiSeq have been deposited at DDBJ (DNA Data Bank of Japan) database with accession numbers of SAMD00042819-SAMD00042826. The hierarchical clustering method using average linkage was used to analyze the relationships between samples. Specific differences in community composition of soil microbes were visualized by use of heat maps using the R package^52^. Chao1 and Shannon index were calculated to estimate the change of microbial diversity.

#### Community and phylogenic analysis using PCR-DGGE

PCR- DGGE analysis was carried out to assess the microbial community of soils by targeting the V3 region of the bacterial 16S rRNA gene. The primer set targeting the V3 region of bacterial 16 S rRNA gene consisted of GC-338f (5′-GCclamp-CACGGGGGGACTCCTACGGGAGGCAGCAG-3′) (GC clamp = CGCCCGCCGCGCGCGGCGGGCGGGGC GGGGGCACGGGGGG) and 518r (5′-ATTACCGCGGCTGCTGG-3′). The PCR amplification and DGGE were performed as per the conditions based on our previous studies^48^. After separation by DGGE, DNA of bands was extracted by using of Poly gel extraction kits (CWBiotech, Beijing, China). 16S rRNA gene fragments were amplified by PCR and then sequenced by ABI Prism 3730XL automated fluorescence sequencer (Applied Biosystems, Foster City, CA, USA). All sequences were compared to those in the GenBank database (http://www.ncbi.nlm.nih.gov). The nucleotide sequences reported in this paper have been deposited in the European Nucleotide Archive database under accession numbers LN649240 to LN649254 (16S rRNA gene DGGE band sequences).

#### Real-time qPCR test of degrading genes with SYBR green I

Three genes *AlkB*, *Nah*, and *Phe* involved in degradation of oil were quantified by real-time qPCR method. In order to minimize effects of environmental factors, numbers of copies of genes were normalized to total number of copies of 16S rRNA genes. Quantification of target genes by qPCR were carried out on a BioRad CFX96 (Hercules, USA) with a C1000 thermal cycler iCycler. Primers for quantification of target genes and related references were given in Supplementary Table S6. Each reaction was performed in a 25 μL volume containing 12.5 μL of qPCR super mix, 0.5 μL of passive reference dye, 0.2 µM of each primer, and 1 μL of DNA template. Cycling conditions were as follows: hold for 30 s at 94 °C followed by 40 cycles of denaturing at 94 °C for 5 s, annealing temperature of target gene primers were set as in Supplementary Table S6 for 15 s, extension at 72 °C for 10 s; hold at 55 °C for 30 s; melting curves were obtained at 55 to 95 °C at a 0.5% heating rate.

### Data availability

Sequences data of amplicons from Illumina HiSeq have been deposited at DNA Data Bank of Japan database (http://www.ddbj.nig.ac.jp/) with accession numbers of SAMD00042819-SAMD00042826. The 16S rRNA gene DGGE band sequences reported in this paper have been deposited in the European Nucleotide Archive database (http://www.ebi.ac.uk/ena) under accession numbers LN649240 to LN649254. A supplement data of this article was represented in Supplemental Material. The Supplement related to this article is available online.

### Statistical analyses

Statistical comparisons of metabolic gene abundances were conducted by SPSS 19.0 for Windows (IBM, Chicago, IL, USA) using nonparametric one-way ANOVA and Kruskal-Wallis Multiple Comparison Z test with Bonferroni adjustment. Redundancy analysis (RDA) were conducted by CANOCO 5.0 for Windows (Microcomputer Power Inc., Ithaca, NY).

## Electronic supplementary material

Supplementary Information
