# Supplementary material for: Aerobic degradation of crude oil by microorganisms in soils from four geographic regions of China
Source: Sci Rep. 2017 Nov 1;7:14856. doi: 10.1038/s41598-017-14032-5 (PMC5665864; doi:10.1038/s41598-017-14032-5)
Supplement: Supplementary file 1 — Supplementary Information [file 41598_2017_14032_MOESM1_ESM.pdf]

Aerobic degradation of crude oil by microorganisms in soils from four geographic regions of China

Qinglong Liu [1], Jingchun Tang *[1,2,3], Kai Gao [4], Ranjit Gurav [1], John P Giesy [5,6,7,8,9]

[1] College of Environmental Science and Engineering, Nankai University, Tianjin 300071, China

[2] Tianjin Engineering Center of Environmental Diagnosis and Contamination Remediation, Tianjin 300071, China

[3] Key Laboratory of Pollution Processes and Environmental Criteria (Ministry of Education), Tianjin 300071, China

[4] Tianjin Academy of Environmental Sciences, Tianjin 300191, China

[5] Toxicology Centre, University of Saskatchewan, Saskatoon, Saskatchewan, Canada

[6] Department of Veterinary Biomedical Sciences, University of Saskatchewan, Saskatoon, Saskatchewan, Canada

[7] School of Biological Sciences, University of Hong Kong, Hong Kong, SAR, China

[8] State Key Laboratory of Pollution Control and Resource Reuse, School of the Environment, Nanjing University, Nanjing, People's Republic of China

[9] Department of Biology, Hong Kong Baptist University, Hong Kong, SAR, China

* Corresponding author: Jingchun Tang

Tel.: +86-22-83614117, Fax: +86-22-83614117

E-mail address: tangjch@nankai.edu.cn

Table S1. Summary of sizes of libraries of sequences for operational taxonomic units (OTUs), and diversity indices based on 16S rRNA gene sequences during incubations of soils in microcosms.

Samples	Number of sequences	Number of OTUs	Ace	Chao I richness	Shannon(H')	Simpson	Coverage (%)
DG0d	5777	247	262	263	3.58	0.0347	99.48
DG112d	7872	195	239	238	3.36	0.0723	99.39
SS0d	10029	187	236	240	1.72	0.4767	99.46
SS112d	11802	114	140	135	2.43	0.1643	99.76
NE0d	12297	270	302	312	4.16	0.0643	99.61
NE112d	10673	94	120	121	2.88	0.1904	99.76
XM0d	11238	123	140	138	2.64	0.1562	99.78
XM112d	13854	139	150	152	3.05	0.0874	99.86

Calculations based on the operational taxonomic units (OTUs) determined at an evolutionary distance of 0.03.

Table S2. Results of sequencing of DGGE bands.

Sequence	Homologous strains	Identity	accession number in ENA
D0-1	*Marinobacter*	99%	LN649243
D0-2	Uncultured *alpha proteobacterium* sp.	100%	LN649242
D0-3	Uncultured *Rhodanobacter* sp.	98%	LN649253
D8-1	Uncultured *Rhodanobacter* sp.	99%	LN649253
D16-1	*Marinobacter*	99%	LN681343
D16-2	*Brevundimonas* sp.	100%	LN681344
S0-1	*Pseudomonas* sp.	99%	LN649240
S1-1	*Streptomyces* sp.	100%	LN649246
S1-2	Uncultured bacterium	98%	LN649248
S16-1	Uncultured *alpha proteobacterium* sp.	98%	LN681346
N0-1	*Pseudomonas* sp.	99%	LN649240
N4-1	*Burkholderia* sp.	100%	LN649249
N8-1	Uncultured *alpha proteobacterium* sp.	100%	LN649252
N8-2	Uncultured *Rhodanobacter* sp.	99%	LN649253
X0-1	Uncultured bacterium	98%	LN649244
X16-1	*Brevundimonas* sp.	100%	LN681344
X16-2	*Burkholderia* sp.	100%	LN649249

Table S3. Shannon-Weiner index quantified based on the DGGE profile.

Samples	0d		7d		28d		56d		112d	
	CK	Crude oil	CK	Crude oil	CK	Crude oil	CK	Crude oil	CK	Crude oil
DG	3.23	3.4	3.13	2.86	3.33	3.38	3.45	3.51	3.44	3.16
SS	2.24	2.16	2.15	2.03	2.99	3.12	2.85	2.96	2.83	3.13
NE	3.85	3.36	3.39	2.52	3.22	2.74	3.46	2.53	3.43	2.66
XM	2.92	2.65	2.06	2.78	2.78	3.16	2.65	2.49	2.86	2.97

Table S4. Absolute numbers of copies of oil-degrading genes in soils from four regions of China.

Samples		DG	SS	NE	XM
Abundance of *AlkB* (copies/g, dm soil)	0d	$(2.75\pm0.40)\times10^{4}$	$(3.44\pm0.13)\times10^{5}$	$(5.42\pm0.31)\times10^{5}$	$(3.44\pm0.53)\times10^{4}$
	7d	$(2.69\pm0.65)\times10^{5}$	$(4.23\pm0.12)\times10^{5}$	$(6.15\pm0.25)\times10^{5}$	$(5.57\pm0.37)\times10^{4}$
	28d	$(1.51\pm0.46)\times10^{6}$	$(3.40\pm0.57)\times10^{7}$	$(3.23\pm0.21)\times10^{7}$	$(2.90\pm0.35)\times10^{6}$
	56d	$(2.73\pm0.26)\times10^{6}$	$(5.79\pm0.43)\times10^{7}$	$(6.33\pm0.42)\times10^{7}$	$(1.70\pm0.53)\times10^{7}$
	112d	$(2.30\pm0.16)\times10^{6}$	$(6.29\pm0.32)\times10^{7}$	$(7.39\pm0.34)\times10^{7}$	$(2.48\pm0.32)\times10^{7}$
Abundance of *Nah* (copies/g, dm soil)	0d	$(4.62\pm0.23)\times10^{3}$	$(1.34\pm0.26)\times10^{3}$	$(1.22\pm0.42)\times10^{3}$	$(1.33\pm0.39)\times10^{3}$
	7d	$(2.83\pm0.53)\times10^{4}$	$(3.72\pm0.43)\times10^{4}$	$(2.26\pm0.51)\times10^{4}$	$(7.85\pm0.57)\times10^{3}$
	28d	$(4.34\pm0.57)\times10^{4}$	$(5.37\pm0.35)\times10^{5}$	$(4.31\pm0.34)\times10^{5}$	$(2.95\pm0.43)\times10^{5}$
	56d	$(6.71\pm0.63)\times10^{4}$	$(7.53\pm0.72)\times10^{5}$	$(6.21\pm0.48)\times10^{5}$	$(3.54\pm0.32)\times10^{5}$
	112d	$(1.29\pm0.43)\times10^{5}$	$(5.36\pm0.64)\times10^{6}$	$(4.23\pm0.73)\times10^{6}$	$(6.42\pm0.55)\times10^{5}$
Abundance of *Phe* (copies/g, dm soil)	0d	$(2.39\pm0.36)\times10^{3}$	$(8.12\pm0.55)\times10^{3}$	$(1.31\pm0.12)\times10^{3}$	$(1.53\pm0.19)\times10^{3}$
	7d	$(1.68\pm0.25)\times10^{4}$	$(1.39\pm0.55)\times10^{5}$	$(9.40\pm0.24)\times10^{4}$	$(2.36\pm0.17)\times10^{3}$
	28d	$(5.54\pm0.54)\times10^{4}$	$(9.67\pm0.25)\times10^{5}$	$(1.30\pm0.97)\times10^{6}$	$(9.40\pm0.13)\times10^{4}$
	56d	$(8.91\pm0.32)\times10^{4}$	$(3.12\pm0.75)\times10^{6}$	$(4.47\pm0.42)\times10^{6}$	$(4.72\pm0.42)\times10^{5}$
	112d	$(1.14\pm0.47)\times10^{5}$	$(2.99\pm0.23)\times10^{6}$	$(3.49\pm0.42)\times10^{6}$	$(5.32\pm0.26)\times10^{5}$
Abundance of 16S rRNA (copies/g, dm soil)	0d	$(6.52\pm0.34)\times10^{7}$	$(2.85\pm0.54)\times10^{8}$	$(2.57\pm0.48)\times10^{8}$	$(1.52\pm0.29)\times10^{7}$
	7d	$(4.34\pm0.12)\times10^{7}$	$(2.65\pm0.21)\times10^{8}$	$(9.40\pm0.64)\times10^{8}$	$(1.10\pm0.22)\times10^{7}$
	28d	$(7.50\pm0.44)\times10^{7}$	$(1.14\pm0.45)\times10^{8}$	$(1.30\pm0.37)\times10^{9}$	$(1.15\pm0.23)\times10^{8}$
	56d	$(9.18\pm0.37)\times10^{7}$	$(1.94\pm0.55)\times10^{9}$	$(4.47\pm0.42)\times10^{9}$	$(5.07\pm0.32)\times10^{8}$
	112d	$(8.92\pm0.48)\times10^{7}$	$(1.22\pm0.36)\times10^{9}$	$(3.49\pm0.31)\times10^{9}$	$(6.43\pm0.46)\times10^{8}$

Table S5. Sequences, sizes of amplicons, and annealing temperatures of primer sets for qPCR.

Degradation genes	Proteins targeted		Sequence(5’-3’)	Amplicon size (bp)	PCR annealing temperature(℃)	Reference
AlkB	Alkane monooxygenases	AlkB-F	AACTACMTCGARCAYTACGG	100	50	[1]
		AlkB-R	TGAMGATGTGGTYRCTGTTCC			
NahAc	Naphthalene dioxygenase	NahAc-F	ACTTGGTTCCGGAGTTGATG	136	57	[2]
		NahAc-R	CAGGTCAGCATGCTGTTGTT			
Phe	Phenol monooxygenase	Phe-F	GTGCTGAC(C/G)AA(C/T)CTG(C/T)TGTTC	206	49	[3]
		Phe-R	CGCCAGAACCA(C/T)TT(A/G)TC			
16S rDNA	Conservative region	16S-F	CGGTGAATACGTTCYCGG	140	55	[4]
		16S-R	CGGTGAATACGTTCYCGG			

References

1. Powell, S.M., Bowman, J.P., Ferguson, S.H. & Snape, I. The importance of soil characteristics to the structure of alkane-degrading bacterial communities on sub-Antarctic Macquarie Island. *Soil Biol Biochem* **42**, 2012-2021 (2010).
2. Park, J.-W. & Crowley, D.E. Dynamic changes in *nahAc* gene copy numbers during degradation of naphthalene in PAH-contaminated soils. *Appl Microbiol Biot* **72**, 1322-1329

(2006).
3. Baldwin, B.R., Nakatsu, C.H. & Nies, L. Detection and enumeration of aromatic oxygenase genes by multiplex and real-time PCR. *Appl Environ Microbiol* **69**, 3350-3358 (2003).
4. Suzuki, M.T., Taylor, L.T. & Delong, E.F. Quantitative analysis of small-subunit rRNA genes in mixed microbial populations via 5'-nuclease assays. *Appl Environ Microbiol* **66**, 4605-4614 (2000).

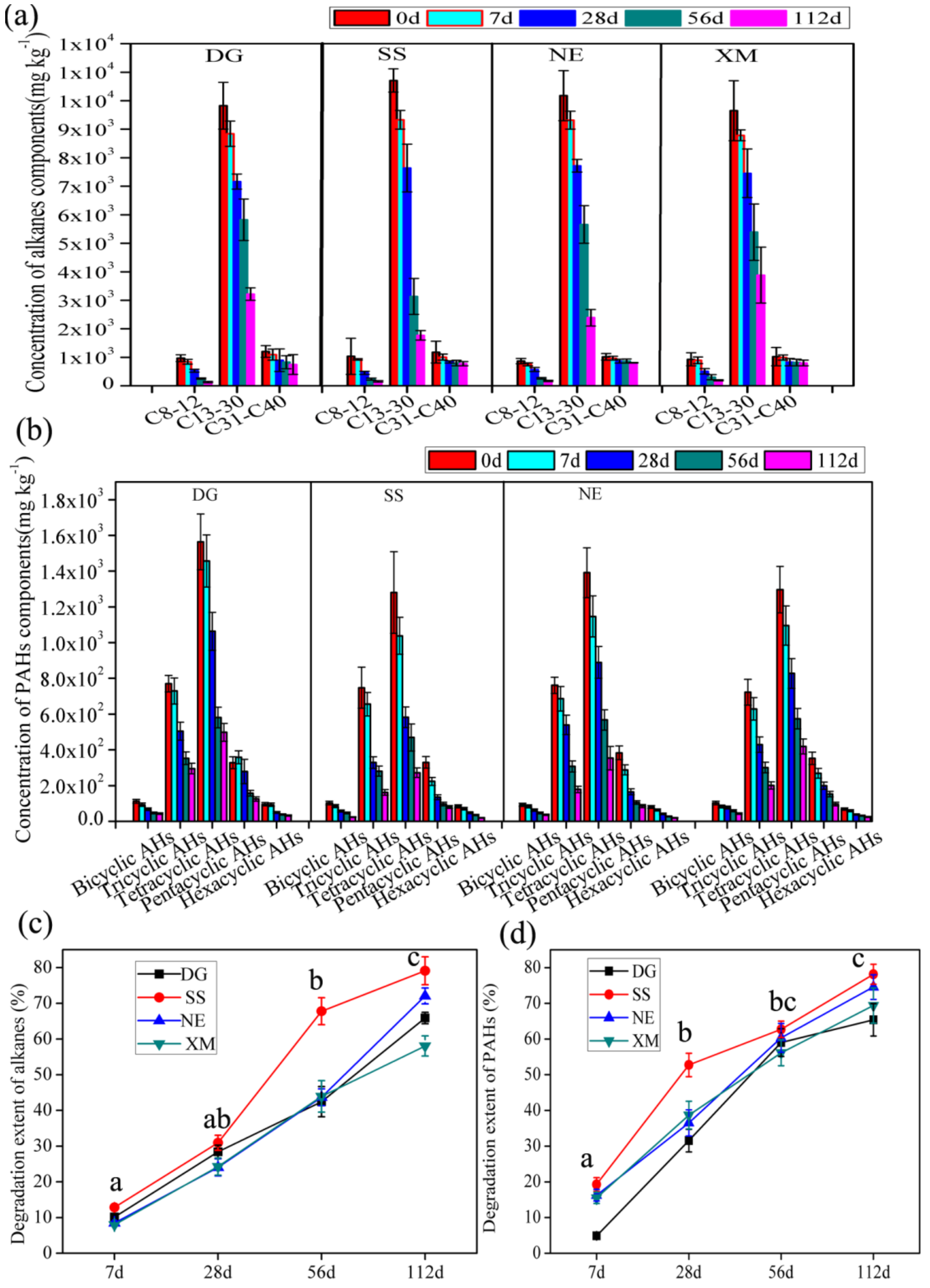

Figure S1. Residual concentrations and degradation of alkanes (C8-C40) and PAHs in soils quantified by use of GC-MS. (a) Concentrations of shorter-chain alkanes (C8-C12), medium-chain alkanes (C13-C30), and longer-chain alkanes (C31-C40). (b) Concentration of bicyclic, tricyclic, tetracyclic, pentacyclic, and hexacyclic PAHs. (c) Degradation extent of shorter-chain alkanes (C8-C12), medium-chain alkanes (C13-C30), and longer-chain alkanes (C31-C40). (d) Degradation extent of bicyclic, tricyclic, tetracyclic, pentacyclic, and hexacyclic PAHs.

Letters on the graph indicate a significant difference among different sampling times at $p < 0.05$ according to Duncan's multiple range tests of One-Way ANOVA.

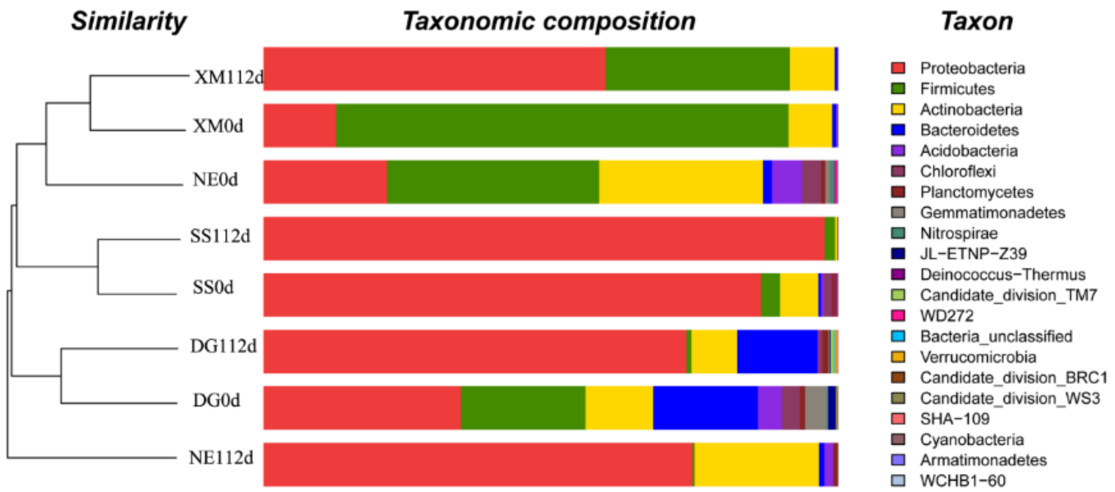

Figure S2. Results of cluster analysis and taxonomic composition of the microbial communities based on HTS. Mean proportions of bacterial phyla in studied soil groups. Phyla representing more than 2% of the bacterial community are summarized.

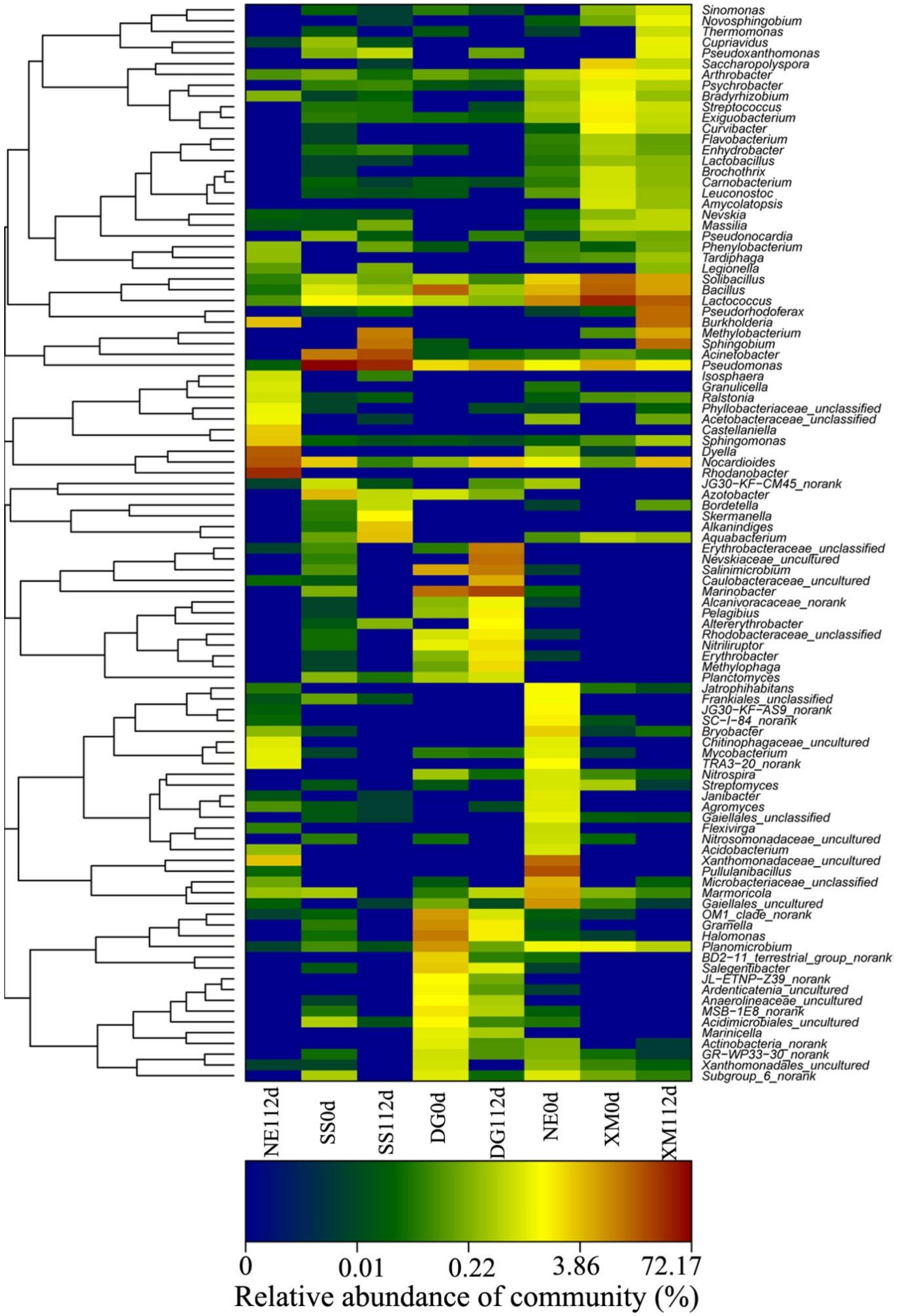

Figure S3. Heat map calculated for the total reads representing restructuring of microbial communities in soils from four geographic regions of China.

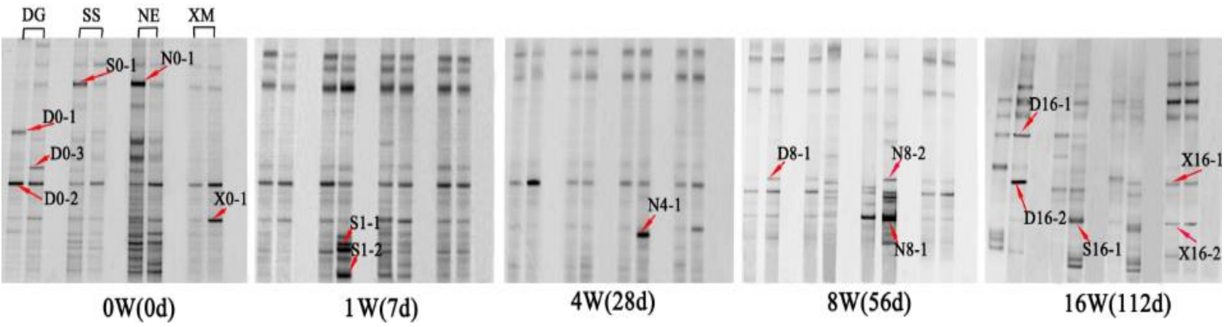

Figure S4. DGGE profile of soils from four typically geographical regions. DGGE bands collected from four soils were of the same order of magnitude as the first picture. In each soil, two bands represented blank control and with addition of 5% (by weight) crude oil, respectively. DG: Dagang Oilfield; SS: Sea Sand; NE: Northeast China; XM: Xiamen.

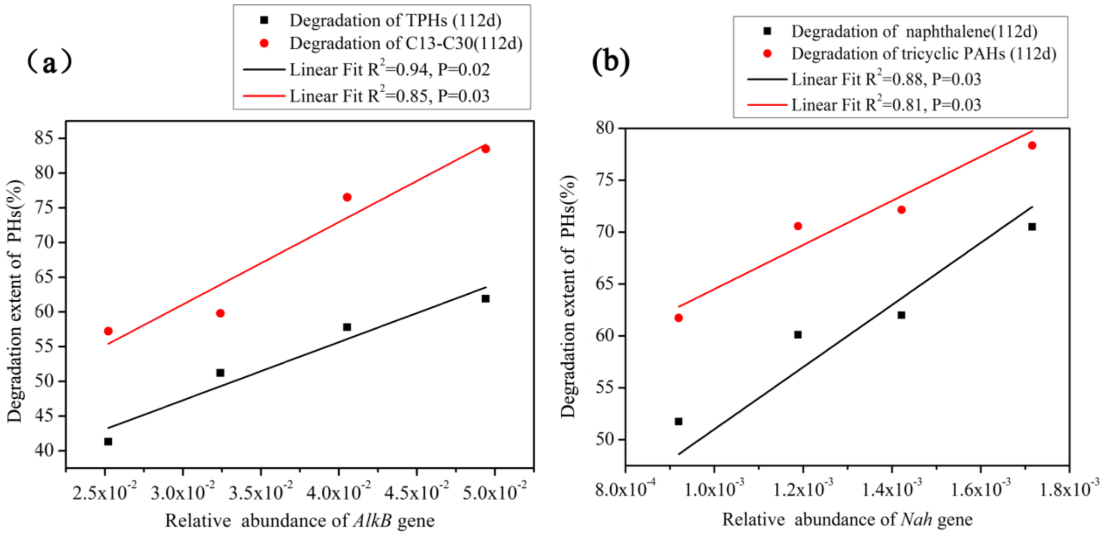

Figure S5. Pairwise correlations between metabolic gene abundances and PHs degradation. (a) Correlation between relative abundances of *AlkB* gene and oil degradation; (b) Correlation between *Nah* gene abundances and oil degradation.

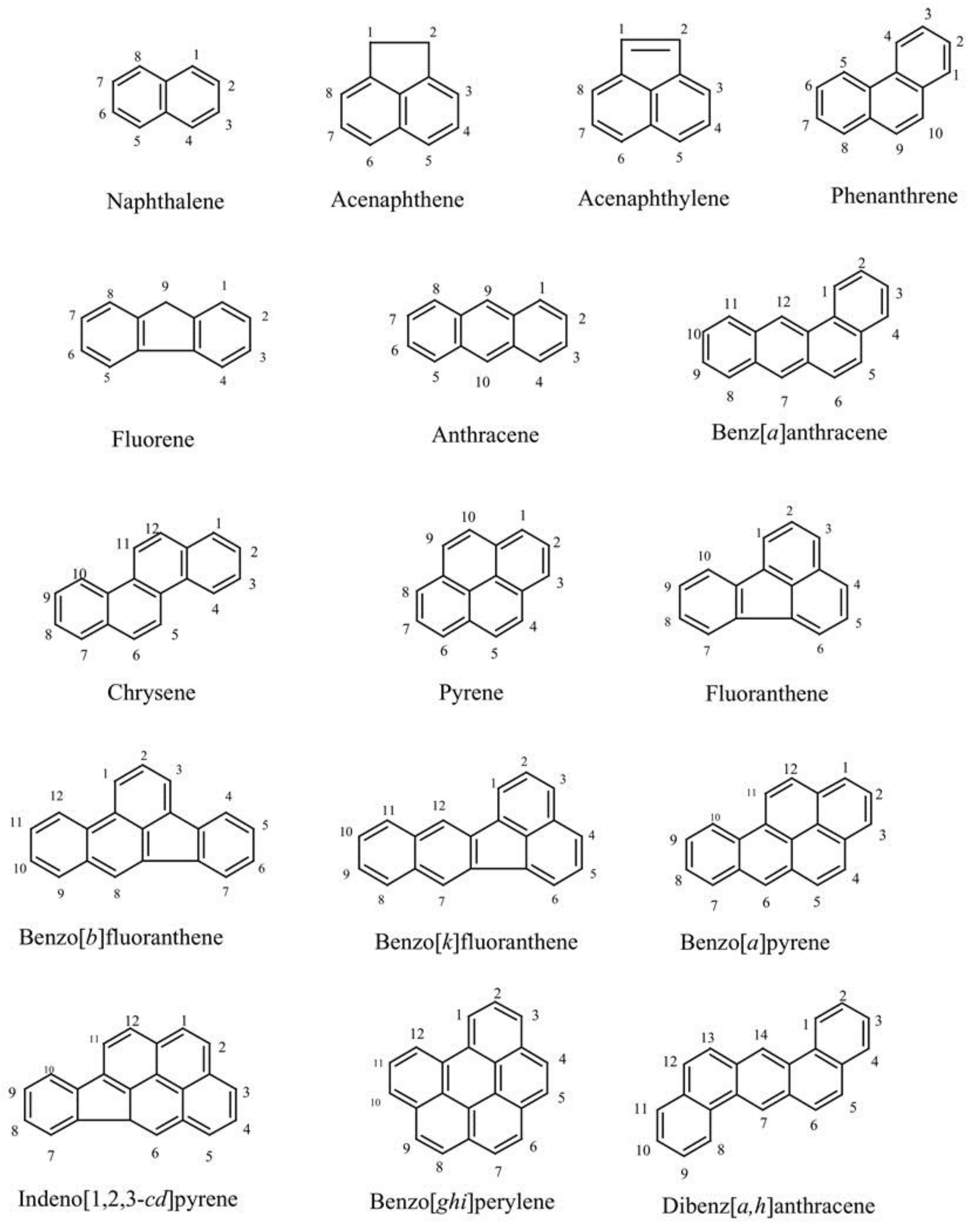

Figure S6. Structures and nomenclatures of the 16 PAHs on the EPA priority pollutant list.
